# Machine Learning-Based Prediction of Impulse Control Disorders in Parkinson’s Disease From Clinical and Genetic Data

**DOI:** 10.1109/OJEMB.2022.3178295

**Published:** 2022-05-27

**Authors:** Johann Faouzi, Samir Bekadar, Fanny Artaud, Alexis Elbaz, Graziella Mangone, Olivier Colliot, Jean-Christophe Corvol

**Affiliations:** Sorbonne Université, Institut du Cerveau - Paris Brain Institute - ICM, CNRS, Inria, Inserm, AP-HPHôpital de la Pitié Salpêtriére 27102 F-75013 Paris France; Department of NeurologyParis Brain Institute, Inserm, CNRS, Sorbonne Université, Assistance Publique Hôpitaux de Paris, Centre d’Investigation Clinique Neurosciences, Hôpital Pitié-Salpêtrière 27102 F-75013 Paris France; Université Paris-Saclay, UVSQ, Université Paris-Sud, Inserm, Gustave Roussy, Équipe “Exposome et Hérédité”, CESP 46657 94807 Villejuif France

**Keywords:** Impulse control disorders, machine learning, Parkinson’s disease, precision medicine

## Abstract

*Goal*: Impulse control disorders (ICDs) are frequent non-motor symptoms occurring during the course of Parkinson’s disease (PD). The objective of this study was to estimate the predictability of the future occurrence of these disorders using longitudinal data, the first study using cross-validation and replication in an independent cohort. *Methods:* We used data from two longitudinal PD cohorts (training set: PPMI, Parkinson’s Progression Markers Initiative; test set: DIGPD, Drug Interaction With Genes in Parkinson’s Disease). We included 380 PD subjects from PPMI and 388 PD subjects from DIGPD, with at least two visits and with clinical and genetic data available, in our analyses. We trained three logistic regressions and a recurrent neural network to predict ICDs at the next visit using clinical risk factors and genetic variants previously associated with ICDs. We quantified performance using the area under the receiver operating characteristic curve (ROC AUC) and average precision. We compared these models to a trivial model predicting ICDs at the next visit with the status at the most recent visit. *Results:* The recurrent neural network (PPMI: 0.85 [0.80 – 0.90], DIGPD: 0.802 [0.78 – 0.83]) was the only model to be significantly better than the trivial model (PPMI: ROC AUC = 0.75 [0.69 – 0.81]; DIGPD: 0.78 [0.75 – 0.80]) on both cohorts. We showed that ICDs in PD can be predicted with better accuracy with a recurrent neural network model than a trivial model. The improvement in terms of ROC AUC was higher on PPMI than on DIGPD data, but not clinically relevant in both cohorts. *Conclusions:* Our results indicate that machine learning methods are potentially useful for predicting ICDs, but further works are required to reach clinical relevance.

## I. Introduction

Although Parkinson’s disease (PD) is mostly known for its motor symptoms, numerous non-motor symptoms have been reported to occur during the course of the disease [1]. Impulse control disorders (ICDs) are psychiatric disorders characterized by the failure to resist an impulse, and unsuccessful attempts to control specific behaviors [2]. ICDs and related disorders are frequent in PD with a prevalence ranging from 15--20% in cross-sectional studies [3], [4], an incidence estimated to around 10% per year [5], [6], and a cumulative incidence reaching almost 50% after 5 years of disease duration in longitudinal studies [5]. PD patients with disease duration greater than 5 years are also subject to these disorders [7]. The four most common ICDs in PD are pathological gambling, compulsive eating, hypersexuality, and compulsive shopping, but other frequent ICDs include punding and hobbyism, and the prevalence of each ICD, in particular pathological gambling, highly varies between different cultures [8]. ICDs are associated with reduced quality of life, strained interpersonal relationships, increased caregiver burden, and require prompt addressing [9]–[11]. Several case reports suggest that partial and total discontinuations of dopamine agonist (DA) treatment leads to a resolution of ICDs [12], [13].

Many factors have been associated with ICDs in PD, including socio-demographic, clinical and genetic biomarkers [14]. In particular, men tend to develop more pathological gambling and hypersexuality disorders while women develop more compulsive buying and eating disorders [15]. A younger age has been associated with ICDs in PD in numerous studies [4], [16]–[18]. Anxiety [18]–[20], depression [16], [20], and rapid eye movement (REM) sleep behavior disorders [21], [22] have also been correlated to ICDs. Dopamine replacement therapy has been shown to be the main risk factor for ICD. Both levodopa and dopamine agonists have been associated with ICDs, but with a stronger and higher association with dopamine agonists. Finally, associations between ICDs and several single-nucleotide polymorphisms (SNPs) in dopamine signaling pathway genes have been suggested [23]–[28].

The predictive performance of these factors altogether has been underexplored. Only three studies reported predictions at the patient level [24], [29], [30]. In all three studies, authors trained a logistic regression using clinical and genetic data, and measured its predictive performance using the area under receiver operating characteristic (ROC) curve (ROC AUC). None of these studies had cross-validation or a replication cohort, altering the confidence in the reported performance [31].

Our main objective was to predict ICDs from clinical and genetic data using machine learning approaches. We utilized two longitudinal cohorts to train and cross-validate the models on one cohort, but also assess the generalization capability of these models on the other cohort. The objective was to predict the risk of ICDs at the next visit, knowing the clinical history of the patient and their genotyping data.

## II. Materials and Methods

### A. Populations

We used data from two research cohorts: the Parkinson’s Progression Markers Initiative (PPMI) database and the Drug Interaction With Genes in Parkinson’s Disease (DIGPD) study.

PPMI (https://www.ppmi-info.org) is a multicenter observational clinical study using advanced imaging, biologic sampling, and clinical and behavioral assessments to identify biomarkers of PD progression [32]. Data was gathered during face-to-face visits every 6–12 months. PD subjects were de novo and drug-naive at baseline. We downloaded the clinical and genetic data from the PPMI database (https://www.ppmi-info.org/data) on the 17th of October, 2019.

DIGPD is a French multicenter longitudinal cohort with annual follow-up of PD patients [5]. Eligible criteria consist in recent PD diagnosis (UK Parkinson’s Disease Society Brain Bank criteria) with disease duration less than 5 years at recruitment. Data was gathered during face-to-face visits every 12 months following standard procedures.

Both studies were conducted according to good clinical practice, obtained approval from local ethic committees and regulatory authorities, and all patients provided informed consent prior to inclusion.

### B. Participants and Clinical Measurements

Inclusion criteria consisted of having:

1) a PD diagnosis,
2) a baseline visit and at least another visit,
3) clinical and genetic data available, and
4) PD medication taken available.

We included socio-demographics and clinical variables that have been associated with ICDs in the literature: age of PD onset, length of follow-up, sex, past ICDs, continuous scales of anxiety, depression and REM sleep, and the motor exam (part III) of the Movement Disorders Society-sponsored revision of the Unified Parkinson’s Disease Rating Scale (MDS-UPDRS). ICDs were assessed at each visit using the Questionnaire for Impulsive-Compulsive Disorders in Parkinson’s Disease - Rating Scale [33] in PPMI, and through semi-structured interviews by a movement disorder specialist in DIGPD. We standardized each feature since some of them were assessed with different scales and because it is a common requirement for most machine learning estimators.

We took into account PD medication with three binary variables corresponding to the main classes of treatment (levodopa, dopamine agonists, others) and we derived more specific variables for dopamine agonists: mean daily, maximum daily and total doses (expressed in levodopa equivalent) and cumulative duration.

### C. Genetic Variants

In absence of genome-wide association study on ICDs in PD, we considered 50 genetic variants selected as previously described: 20 variants from 16 genes involved in dopamine, serotonin, glutamate, norepinephrine and opioid systems and previously associated with ICD in PD or in the general population [23]; 30 additional variants from 10 genes differentially expressed after an acute challenge of levodopa in the striatum in a mouse model of dopamine denervation [34].

Genotyping data were collected using NeuroX [35] arrays in PPMI (267,607 variants measured), and Illumina Multi-Ethnic Genotyping Arrays in DIGPD (1,779,819 variants). We excluded variants with missing rates greater than 2% and variants deviating from Hardy-Weinberg equilibrium ($p < 10^{-8}$). We excluded related individuals (third-degree family relationships), individuals with mismatch between reported sex and genetically determined sex, and individuals with outlying heterozygosity ($\pm$ 3 standard deviation). We imputed missing SNPs using the Michigan Imputation Server [36] for PPMI and the Sanger Imputation Server [37] for DIGPD, using the reference panel of the Haplotype Reference Consortium (release 1.1) [37]. We filtered variants based on their imputation quality ($r^{2} > 0.6$ for PPMI, INFO score $> 0.9$ for DIGPD) in order to only include variants imputed with high quality.

### D. Data Processing

Processing genetic data and extracting variants of interest matching inclusion criteria was performed using the PLINK [38] software. Processing of the different text-like files was performed using the pandas [39] and NumPy [40] Python packages. Missing values were imputed in a forward-fill fashion: for a given subject and a given feature, missing values were imputed using the most recent non-missing value for this subject and this feature. Baseline missing values were imputed using the mean baseline values on the training set. We used this simple approach because only a small percentage of data from a small subset of variables (anxiety, depression, REM sleep, and motor exam) was imputed and because it can be applied to any scale without any training.

### E. Machine Learning Algorithms

We investigated five standard machine learning algorithms implemented in the scikit-learn [41] and XGBoost [42] Python packages: logistic regression, support vector machines with a linear kernel and with a radial basis function kernel [43], [44], random forest [45] and gradient tree boosting [46], [47]. These algorithms expect a fixed number of features as input. In order to deal with varying numbers of visits, we reduced all the previous visits into one “summary” visit using a convex combination. A convex combination is a linear combination such that the weights are all non-negative and sum to one. The weights indicate how much each visit contributes to this “summary” visit. A weight of 1 for the first visit means that the “summary” visit is simply the baseline visit, while a weight of 1 for the latest visit means that the “summary” visit is simply the most recent visit. One can also give uniform weights, so that each visit contributes equally to this “summary” visit, or higher weights to most recent visits if they are assumed to be more important than older visits.

**Fig. 1.**
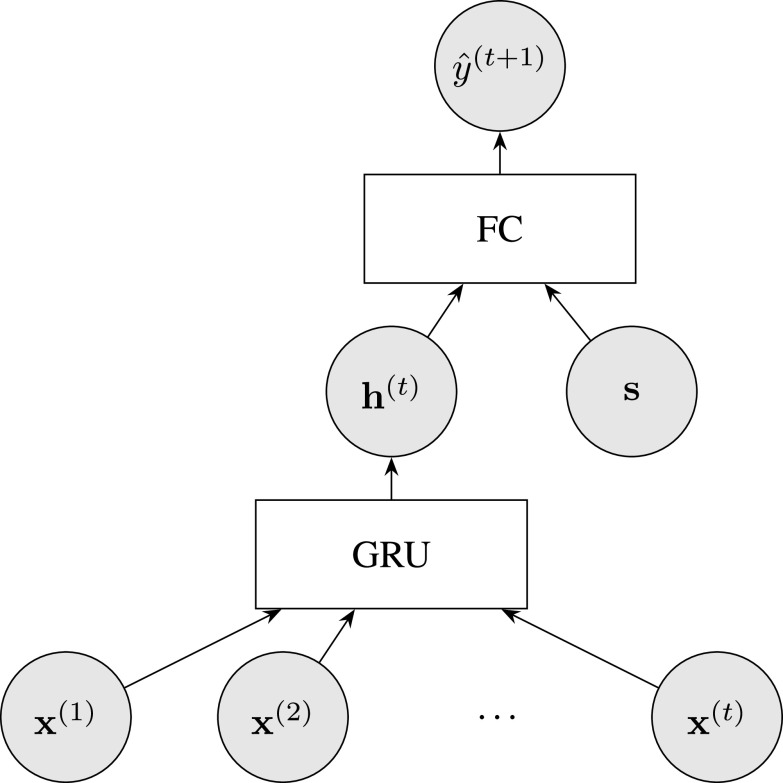
Architecture of the recurrent neural network. The clinical features assessed at several visits, denoted as $(\mathbf {x}^{(1)}, \ldots, \mathbf {x}^{(t)})$, are used as input of the Gated Recurrent Unit (GRU). The GRU extracts information from these clinical features into a vector $\mathbf {h}^{(t)}$. This vector and the time-independent variables, namely the socio-demographic and genetic data denoted as $\mathbf {s}$, are used as input of a Fully Connected (FC) layer followed by a sigmoid activation function, returning the probability of having an impulse control disorder at the next visit, denoted as $\hat{y}^{(t+1)}$.

As the prediction task is longitudinal, we also investigated the use of recurrent neural networks. Recurrent neural networks are a class of artificial neural networks dedicated to sequential data. We employed a simple architecture (Fig. 1) with a Gated Recurrent Unit [48] to extract information from the clinical measurements, followed by a concatenation of this vector with the socio-demographic and genetic data, followed by a Fully Connected layer with a sigmoid activation function. We used the PyTorch [49] Python package to build and train the recurrent neural network.

The recurrent neural network model is the only algorithm that learns to transform the variable-length sequence of inputs into a vector. All the machine learning models are trained end-to-end and there is no separate estimation of the $\mathbf {h}^{(t)}$ vector in the recurrent neural network model.

All the machine learning models are trained end-to-end and take as input clinical (age of PD onset, sex, past ICDs, anxiety, depression, REM sleep, types of PD medication and specific variables for the use of dopamine agonists, as described in Section II-B) and genetic data (as described in Section II-C), except in Section III-C in which we compare the models with the same machine learning algorithms trained with clinical data only.

Since the task is longitudinal, we defined a trivial model as the one that predicts the ICD status of a patient at the next visit with the ICD status at the most recent visit.

### F. Cross-Validation

**Fig. 2.**
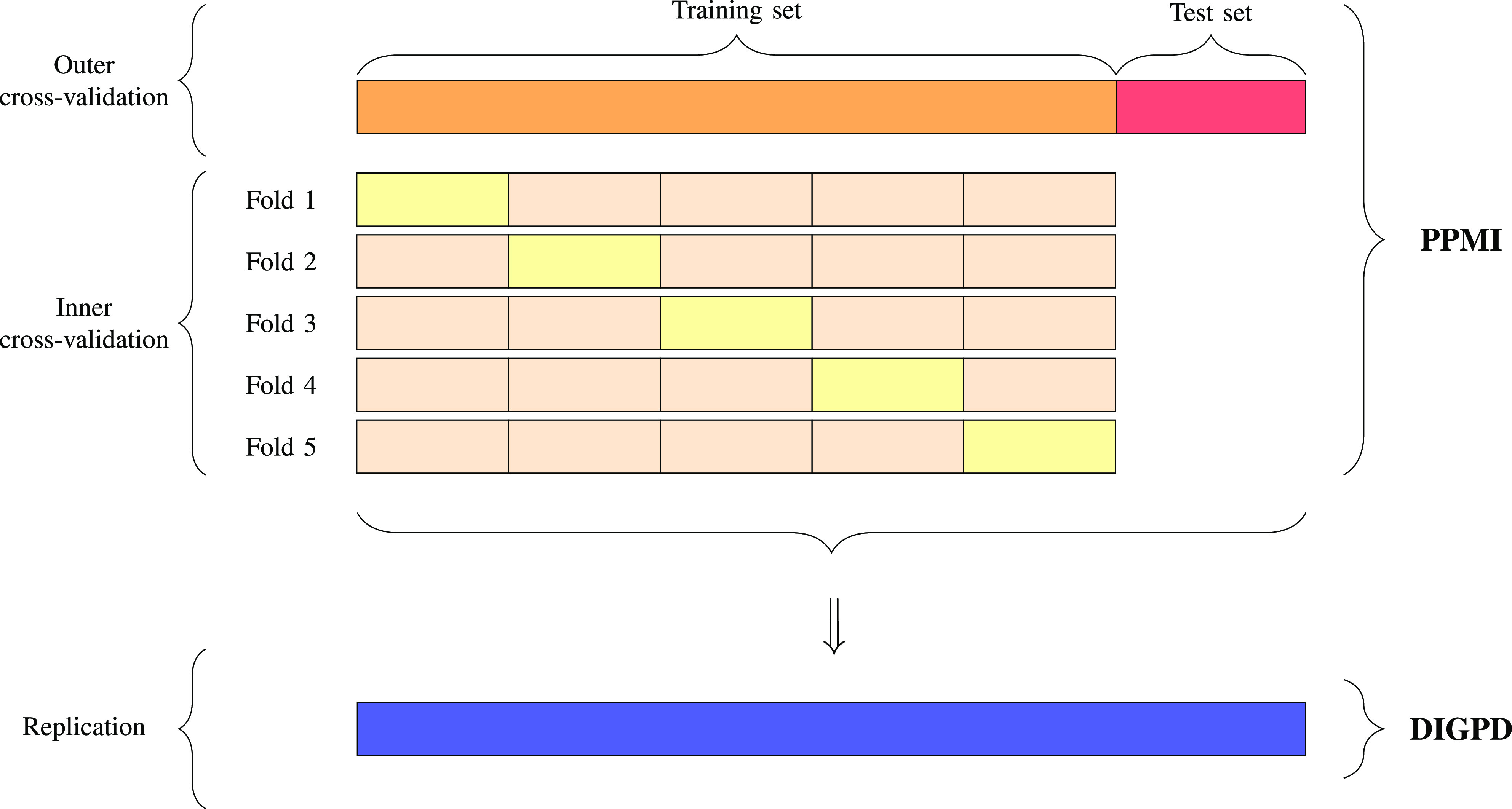
Cross-validation procedure. We employed a nested cross-validation procedure on the PPMI cohort. In the outer loop, we split the PPMI subjects into training and test sets, while the inner loop was a 5-fold subject-level cross-validation to optimize the hyper-parameters of the model. The model with the optimal values for the hyper-parameters was evaluated on the test set of PPMI and on the whole DIGPD cohort.

We used PPMI as the training (discovery) cohort, and DIGPD as the test (replication) cohort. To unbiasedly estimate the predictive performance of the models, we employed a nested cross-validation procedure that is illustrated in Fig. 2. In the outer loop, we randomly split 80% of the PPMI subjects into the training set and the remaining 20% into the test set. In the inner loop, we performed a 5-fold subject-level cross-validation procedure to optimize the hyper-parameters of the models on the training set. These hyper-parameters control how the algorithms fit the training data. For instance, these hyper-parameters included the type ($\ell _{1}$ or $\ell _{2}$ penalty) and amount ($\lambda$ parameter) of regularization for the linear models. In particular, logistic regression models were regularized. The regularization applies to all the input data (clinical and genetic). After finding the optimal values for the hyper-parameters, each model was evaluated on the test set. Finally, we evaluated the performance of each model on the whole DIGPD cohort.

### G. Statistical Analysis

Baseline characteristics in both cohorts were compared with chi-squared tests for categorical variables and t-tests for continuous variables using the SciPy [50] Python package. Predictive performance was mainly evaluated using the area under the receiver operating characteristic curve and average precision (AP). AP summarizes a precision-recall curve as the weighted mean of precisions achieved at each threshold, with the increase in recall from the previous threshold used as the weight. The precision-recall curve is similar to the ROC curve, but plots the precision (positive predictive value) against the recall (sensitivity). The precision-recall curve does not take into account the true negatives, and is particularly useful when the positives are more important than the negatives (false negatives are more serious than false positives). Other metrics included accuracy, balanced accuracy (BA), sensitivity and specificity. ROC and precision-recall curves were plotted using the matplotlib [51] Python package, and all the metrics were computed using the scikit-learn [41] package. Comparison between ROC AUC was measured using the DeLong test [52]. P-values were adjusted for multiple comparisons using Bonferroni correction. We did not perform hypothesis testing to compare AP scores as we were not aware of a relevant statistical test to do so.

## III. Results

### A. Population Characteristics

Out of the 423 PD subjects in PPMI, we excluded 1 subject for not having a baseline visit, 2 for not having medication records and 40 for not having genetic data. Out of the 415 PD subjects in DIGPD, we excluded 27 for having only a baseline visit. No subjects were excluded based on their genetic data. Thus, we included 380 PD subjects from PPMI and 388 PD subjects from DIGPD in our analyses. The 380 PPMI subjects had a total of 2,728 visits, while the 388 DIGPD subjects had a total of 2,101 visits. Since our objective was to predict the occurrence of ICDs at the next visit, the number of observations for a given subject is equal to their number of visits minus 1. Thus, the total number of observations was equal to 2,348 in PPMI and 1,713 in DIGPD.

Clinical characteristics are presented in Table I. Age and sex in both cohorts were not significantly different. PPMI subjects had significantly more visits and smaller intervals between back-to-back visits, as well as longer follow-ups. DIGPD subjects had significantly lower scores in the motor exam of the MDS–UPDRS. The prevalence of ICDs at baseline was significantly higher in DIGPD than in PPMI, as well as their lifetime prevalence. Both differences might be explained by the fact that PD subjects are de novo and drug-naive at baseline in PPMI whereas they are not in DIGPD. Other phenotypes (anxiety, depression, and REM sleep disorders) were not statistically compared due to the different scales used.

**TABLE I:** Baseline Characteristics. For Continuous Variables, Mean $\pm$ Standard Deviation Is Reported. For Binary Variables, the Count for Both Categories is Reported as Well as the proportion of the First Category. Statistical Differences Were Assessed Using Independent t Tests for Continuous Variables and Chi-Squared Tests for Binary Variables. GDS: Geriatric Depression Scale; HAD: Hospital Anxiety and Depression Scale; RBDSQ: Rapid eye movement Sleep Behavior Disorder Screening Questionnaire; STAI: State-Trait Anxiety Inventory

Characteristic	PPMI	DIGPD	$p$-value
Age (in years)	$60.67 \pm 9.71$	$58.99 \pm 9.75$	${1.71\times 10^{-2}}$
Sex (F/M)	$127 / 253$ $(33\%)$	$155 / 233$ $(40\%)$	${7.16\times 10^{-2}}$
Length of follow-up (in years)	$5.86 \pm 1.95$	$4.82 \pm 1.83$	${7.07\times 10^{-14}}$
Number of visits per subject	$7.18 \pm 2.96$	$5.41 \pm 1.66$	${4.21\times 10^{-35}}$
Interval between visits (in years)	$0.95 \pm 0.35$	$1.09 \pm 0.33$	${3.24\times 10^{-39}}$
Anxiety	STAI: $93.55 \pm 7.96$	HAD: $6.82 \pm 3.77$	
Depression	GDS: $5.25 \pm 1.47$	HAD: $4.59 \pm 3.16$	
REM sleep	RBDSQ: $4.17 \pm 2.71$	1/0: $86 / 302 (22\%)$	
MDS-UPDRS III	$20.87 \pm 8.86$	$9.91 \pm 5.33$	${3.77\times 10^{-72}}$
Baseline ICD (1/0)	$42 / 338$ $(13\%)$	$76 / 312$ $(20\%)$	${5.34\times 10^{-3}}$
Lifetime ICD (1/0)	$143 / 237$ $(38\%)$	$192 / 196$ $(49\%)$	${1.12\times 10^{-3}}$

Concerning genetic data, we excluded 1 genetic variant for being a variable number of tandem repeat polymorphism. Furthermore, we excluded 18 SNPs for having too low imputation quality scores. Finally, 31 SNPs were included in our analyses (Supplementary Table 1).

### B. Predictive Performance

**Fig. 3.**
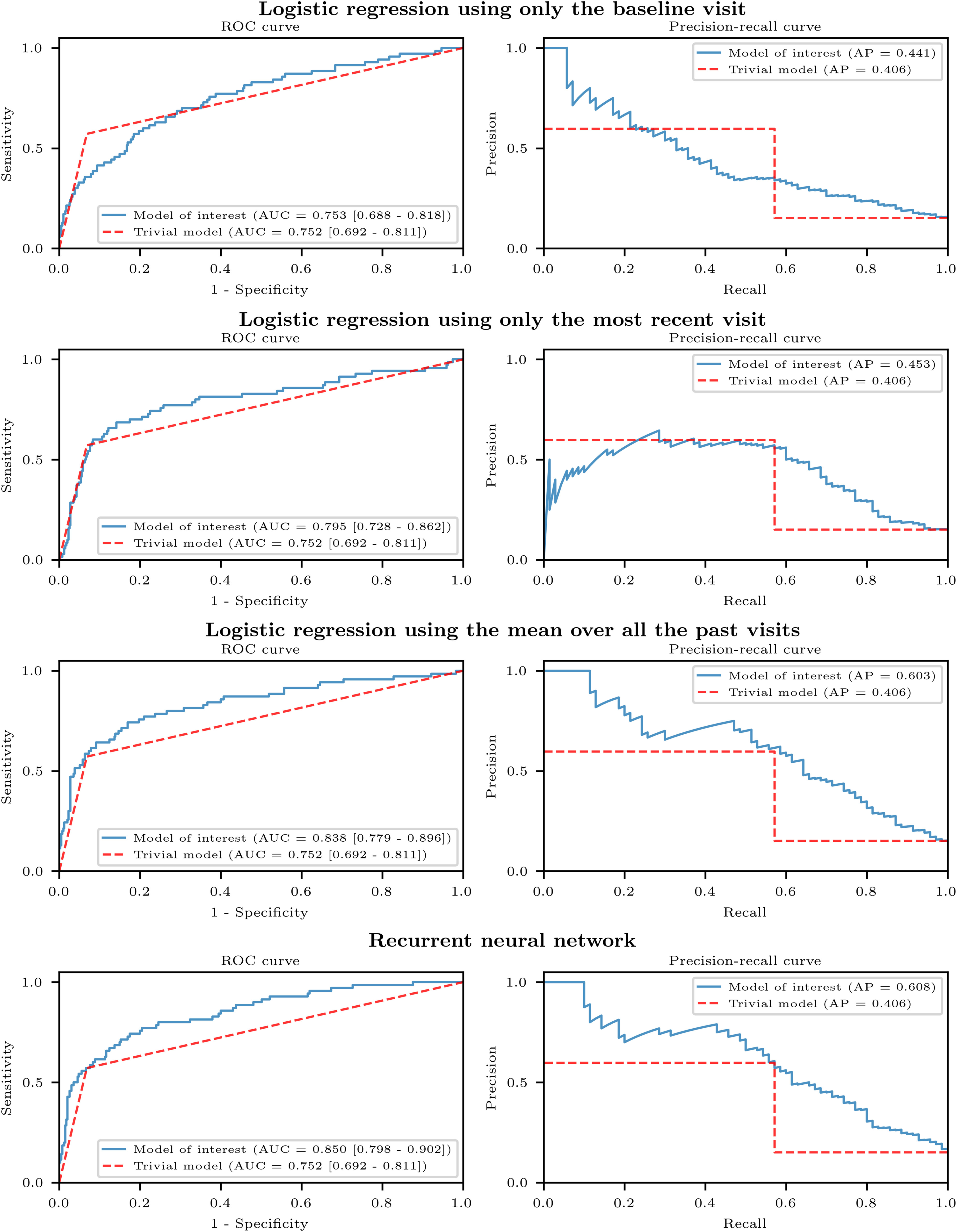
ROC and Precision-recall curves on PPMI. The models, trained on a subset of subjects in PPMI, were evaluated on an independent subset of subjects in PPMI.

**Fig. 4.**
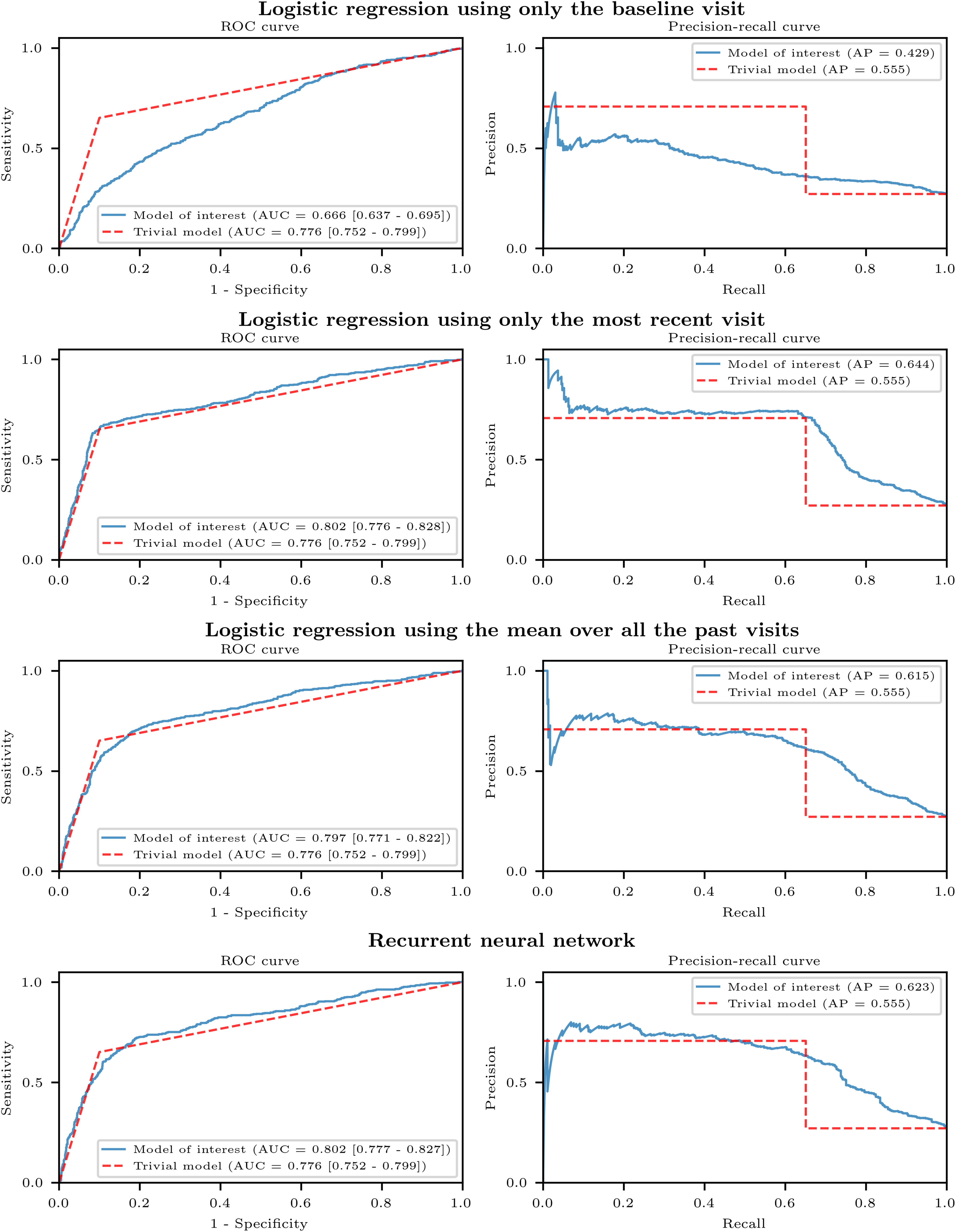
ROC and Precision-recall curves on DIGPD. The models, trained on a subset of subjects in PPMI, were applied on all the subjects in DIGPD.

Table II presents the predictive performance for the five main models: the trivial model, logistic regression models using the baseline visit, the most recent visit, and the mean over all the past visits respectively, and the recurrent neural network model. The logistic regression model using the baseline visit had some of the lowest scores on both cohorts (ROC AUC = 0.75 and AP = 0.44 in PPMI, ROC AUC = 0.67 and AP = 0.43 in DIGPD). The trivial model reached a ROC AUC = 0.75 of in PPMI and of 0.78 in DIGPD. The recurrent neural network yielded the highest scores in PPMI (ROC AUC = 0.85, AP = 0.61), while the logistic regression using the most recent visit yielded the highest scores in DIGPD (ROC AUC = 0.80, AP = 0.64). Fig. 3 and Fig. 4 show the ROC and precision-recall curves for the four main models compared to the trivial model in PPMI and in DIGPD respectively. The recurrent neural network models had sensitivities of 61% and 70% and specificities of 90% and 82% in PPMI and DIGPD respectively, at the default threshold (probability $> 0.5$).

**TABLE II:** Results of the Four Main Models. Predictive Performance for the Five Main Models on Both Cohorts Are Reported

		Trivial model	Logistic regression using only the baseline visit	Logistic regression using only the previous visit	Logistic regression using the mean over past visits	Recurrent neural network
ROC AUC	PPMI	0.752	0.753	0.795	0.838	0.850
		DIGPD	0.776	0.666	0.802	0.797	0.802
Average precision	PPMI	0.406	0.441	0.453	0.603	0.608
		DIGPD	0.555	0.429	0.644	0.615	0.623
Accuracy	PPMI	0.877	0.774	0.819	0.841	0.860
		DIGPD	0.833	0.566	0.592	0.645	0.785
Balanced accuracy	PPMI	0.752	0.691	0.764	0.765	0.759
		DIGPD	0.776	0.603	0.668	0.695	0.758
Sensitivity	PPMI	0.571	0.571	0.686	0.657	0.614
		DIGPD	0.651	0.684	0.838	0.805	0.699
Specificity	PPMI	0.932	0.810	0.843	0.873	0.904
		DIGPD	0.900	0.522	0.499	0.584	0.817

**Fig. 5.**
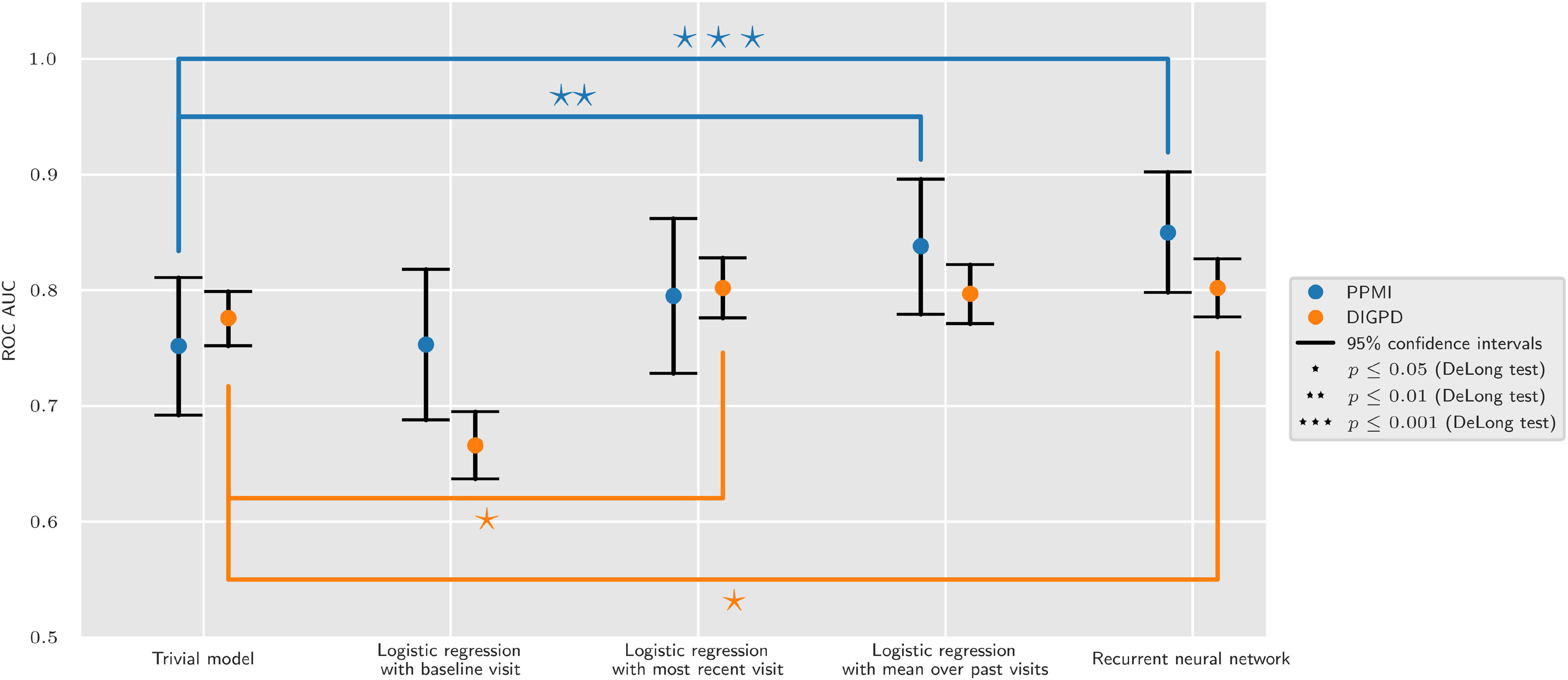
Statistical comparison of ROC AUC for the five main models. 95% confidence intervals were computed using the asymptotic normal distributions of the ROC AUC scores. P-values were computed using the DeLong test. P-values below the 0.05 threshold after adjustment for multiple comparison using Bonferroni correction are highlighted with at least one asterisk: $p \leq 0.05 \, (\star)$, $p \leq 0.01 \, (\star \star)$, $p \leq 0.001 \, (\star \star \star)$.

The recurrent neural network model was the only model to be significantly better than the trivial model on both cohorts, and two logistic regression models were significantly better than the trivial model on one cohort (Fig. 5).

Although AP scores for the three best models were higher in DIGPD than in PPMI, the prevalence of ICDs, computed over all the (patient, visit) pairs, was twice higher in DIGPD than in PPMI (27% in DIGPD, 14% in PPMI). As AP scores of random guess are equal to the prevalence of the positive class, the differences between AP scores in both cohorts should be interpreted with much caution.

The other machine learning algorithms (support vector machines with linear and radial basis function kernels, random forest, and gradient tree boosting) and other reduction approaches (giving positive weights to all the past visits, but higher weights to more recent visits) yielded comparable results (Supplementary Table 2).

To evaluate the impact of the splitting of PPMI into training and test sets on the predictive performance, we repeated the cross-validation procedure 10 times and also evaluated the 10 models on DIGPD. All iterations yielded comparable results (Supplementary Table 3).

To evaluate the impact of the choice of the training and replication cohorts, we used DIGPD as the training cohort and PPMI as the replication cohort and obtained similar results (Supplementary Tables 4 and 5).

### C. Contribution of the Different Features

Since the genetic factors of ICDs in PD are mostly unknown and genotyping data is not usually collected in clinical routine, we investigated the predictive performance of the same algorithms without the genetic variants as input, in order to assess their added value in the models. Table III presents the ROC AUC of the models with and without genetic variants and their statistical comparison. Only one comparison was statistically different: the logistic regression model using the most recent visit had a higher ROC AUC with genetic variants than without genetic variants on DIGPD (ROC AUC = 0.80 with genetic variants, ROC AUC = 0.79 without genetic variants, $p < 0.001$). The genetic variants did not seem to be major contributors to the decision function of the logistic regression models.

**TABLE III:** Statistical Comparison of ROC AUC for the Four Main Models With and Without Genetic Variants. Differences in ROC AUC Between the Models With and Without Genetic Variants Were Assessed With the DeLong Test. Significant Differences After Bonferroni Correction Are Highlighted in Bold Font

		Logistic regression using only the baseline visit	Logistic regression using only the previous visit	Logistic regression using the mean over past visits	Recurrent neural network
PPMI	With genetic variants	0.753 [0.688 – 0.818]	0.795 [0.728 – 0.862]	0.838 [0.779 – 0.896]	0.850 [0.798 – 0.902]
		Without genetic variants	0.751 [0.687 – 0.816]	0.807 [0.745 – 0.868]	0.843 [0.789 – 0.899]	0.845 [0.792 – 0.899]
		$p$-value	0.885	0.0971	0.637	0.451
DIGPD	With genetic variants	0.666 [0.637 – 0.695]	0.802 [0.776 – 0.828]	0.797 [0.771 – 0.822]	0.802 [0.777 – 0.827]
		Without genetic variants	0.682 [0.654 – 0.711]	0.786 [0.758 – 0.813]	0.788 [0.762 – 0.813]	0.803 [0.778 – 0.828]
		$p$-value	0.0143	**0.000252**	0.109	0.757

We also investigated the coefficients of the three logistic regression models with and without genetic variants as input (Supplementary Tables 6 and 7). As the logistic regression model using the baseline visit did not perform better than the trivial model, and the variables for PD medication were all null (PD patients in PPMI are de novo drug-naive at baseline, and the medical history of PD patients in DIGPD was not available before their baseline visit), we only interpreted the other two models. The following features had positive coefficients: sex, past ICDs, depression, REM sleep, motor exam, being on other PD medication than levodopa and dopamine agonists, and maximum dose and cumulative duration of dopamine agonists. On the other hand, the following features had negative coefficients: age, anxiety, being on levodopa, and mean daily and total dose of dopamine agonists. The features corresponding to being on dopamine agonists and time to prediction had coefficients close to zero. The variables with the largest absolute values were past ICDs, and total dose and cumulative duration of dopamine agonists.

## IV. Discussion

To the best of our knowledge, this study is the first one evaluating the predictability of ICDs in PD in an unbiased manner using two longitudinal cohorts, including one independent replication cohort.

Three previous studies reported ROC AUC for a prediction task of ICDs in PD [24], [29], [30]. Kraemmer and colleagues reported ROC AUC of 0.65 [0.58 – 0.73] with clinical variables only and of 0.76 [0.70 – 0.83] with clinical and genetic variables, while Erga and colleagues reported ROC AUC of 0.68 [0.59 – 0.78] with clinical features only and of 0.70 [0.61 – 0.79] with clinical and genetic features, and Jesus and colleagues reported ROC AUC of 0.69 with clinical features only and of 0.80 with clinical and genetic features. However, the methods and prediction tasks were different. Erga and colleagues performed a cross-sectional analysis of 119 PD patients from the Norwegian ParkWest study, Jesus and colleagues also performed a cross-sectional analysis of 353 PD patients recruited from the Movement Disorder Clinic of the University Hospital Virgen del Rocío in Seville, Spain, while Kraemmer and colleagues performed a longitudinal analysis of 276 PD patients from PPMI. In their studies, each patient corresponds to a unique observation, leading to much lower sample sizes. Moreover, all three studies did not use an independent test set nor cross-validation and did not have a replication cohort, which might lead to overly optimistic reported results [31]. By contrast, our study uses cross-validation on the training cohort (PPMI), an independent test set from PPMI and a replication cohort (DIGPD). Results were overall comparable in both cohorts, even though the performance was slightly lower in DIGPD than in PPMI, and the difference with the trivial model was also less substantial, although statistically significant. This may be explained by the fact that both cohorts have different characteristics (de novo drug-naive patients in PPMI, already-treated patients in DIGPD) and some variables (anxiety, depression, REM sleep) were not measured with the same instruments.

The best performing model was the recurrent neural network. It achieved a statistically higher performance than the trivial model in both cohorts. Nevertheless, while the difference in performance was substantial (10 percentage points of ROC AUC) for the PPMI dataset, it was much weaker for DIGPD dataset. This may be partly due to the difficulty of generalizing to a different cohort with different inclusion criteria and different measurement scales. Nevertheless, the results obtained when using DIGPD for training and PPMI for replication (see Supplementary Tables 4 and 5) suggest that prediction may be inherently slightly more difficult in the DIGPD cohort.

The logistic regression coefficients were overall consistent with the literature. For the socio-demographic variables, sex and age have respectively a positive and negative coefficients, in accordance with a younger age and a male sex previously associated with ICDs in PD [15]. Depression, REM sleep and motor exam scores also had positive coefficients, consistent with their positive association [14]. Anxiety scores had negative coefficients although previously reported to be positively associated with ICD. The maximum dose and cumulative duration of dopamine agonists had positive coefficients, confirming the important role of the dose and the duration of dopamine agonist therapy in the risk to develop ICDs in PD [5]. Interestingly, the types of PD medication sparsely contributed to the decision function of the models, with very small coefficients. The mean daily and total doses of dopamine agonists had negative coefficients, although these coefficients were almost null for the mean daily dose. These derived features have rarely been investigated altogether, making the comparison with the literature difficult. It should be noted that the coefficients are estimated altogether and that the logistic regression models were regularized, so interpretation should be performed with caution.

We used features that have been associated with ICDs in PD as input of our models, but there are probably more unknown risk factors to be discovered. In addition, as a class of psychiatric disorders, ICDs are particularly complex, with qualitative environmental factors that might play important roles, are difficult to measure, and are not captured by clinical scales used in PD. Assessment of ICDs may also be noisy (e.g. patients hiding or not aware of their behavior), and thus ICDs are probably less predictable in practice than other comorbidities in PD, such as dementia [53]. Finally, little is known about the genetic factors of ICDs in PD. In our study, the inclusion of genetic variants did not lead to a substantial improvement over clinical data alone for the best performing models. In absence of genome-wide association study and genetic risk scores for ICDs in PD, we used associated genetic variants from candidate gene analyses [23], [24], [29]. As variation in complex traits is caused by numerous genetic variants, such analyses have important limitations and many association studies could not be replicated, particularly in psychiatric conditions like schizophrenia [54]. More studies, in particular genome-wide association studies, are needed to better understand the genetic landscape of ICDs in PD.

Being able to predict ICDs is of critical importance due to their potential medical, financial, and/or legal medical complications. Identifying patients at high risk to develop ICDs at the next visit may lead to changes in the dopaminergic treatment strategy (e.g. decrease the dose of dopamine agonists and increase levodopa) and/or recommend a closer monitoring of behavioral changes by the caregiver. The efficacy of such preventive strategies based on a predictive model remains however to be evaluated. In this perspective, the model may be adapted depending on the relative importance for identifying positives (patients who will develop ICDs) or negatives (patients who will not develop ICDs). The balanced accuracy scores were equal to 76% for the recurrent neural network models in both cohorts, but the sensitivities (61% vs 70%) and specificities (90% vs 82%) differed, which might be explained by the different prevalences in both cohorts. Using the default threshold (probability $> 0.5$) made the models more specific than sensitive, which might be a limitation if finding the positives is more important than the negatives. On the other hand, models being more specific than sensitive might be more relevant if the main objective is to propose treatment changes only to patients who are at strong risk, and avoid unnecessary modifications in more patients. The threshold can still be adjusted depending on the main objective. Prospective studies are required to validate the models and allow their relevance in clinical routine. Another clinically relevant task would be to predict the first onset of ICDs in PD, but is left for future work.

Our study has several limitations. A first important limitation is that the performances of the best models were only marginally better than that of a trivial model on the replication cohort DIGPD. While the difference was statistically significant, its magnitude is too small for the tool to be useful in clinical practice in its current state. They nevertheless set a methodologically solid basis for the development of improved models. Second, as mentioned in Section II-B, the definition of ICDs differ between cohorts. This may partly explain why our machine learning models struggle to generalize in the independent replication cohort. Third, the sample sizes are relatively small, in particular on the test set of PPMI due to the use of cross-validation. Fourth, each observation is a (subject, visit) pair and thus the observations are not independent (the intra-subject observations are not independent, but the inter-subject observations are independent), which could lead to underestimating p-values when assessing the statistical difference between ROC AUC. Fifth, in absence of genome-wide association study and genetic risk scores for ICDs in PD, we used associated genetic variants from candidate gene analyses. Genetic risk scores are more robust estimators of the genetic liability of a phenotype and should be preferred when available [55]. We previously investigated the genetics of ICDs in PD by computing genetic risk scores for other phenotypes and investigating their statistical association with ICDs, but we could not report any statistical association [56]. Sixth, we did not include several risk factors of ICDs in PD as input of the algorithms, such as dyskinesia [57] and sleep disorders other than REM sleep. Nonetheless, the added value of known risk factors that were not included remains to be proven, as these risk factors were discovered in univariate analyses. Moreover, adding more features as input of machine learning algorithms does not necessarily lead to better predictive performance due to the correlation between the features and the risk of overfitting.

## V. Conclusion

Our study shows the feasibility of prediction of impulse control disorders in Parkinson’s disease. Nevertheless, the improvements obtained compared to a trivial model are not sufficient to support clinical utility at this stage of research. Nonetheless, our study highlights a sound methodology and sets a baseline that future studies can compare to. Further studies including other risk factors and investigating the first onset of ICDs are required to obtain clinically relevant models.

## Supplementary Materials

We list the different reduction approaches that we investigated and the genetic variants included in our analyses. We also provide additional experiments with other machine learning algorithms and other reduction approaches, with several cross-validation runs, and the same experiments but with swapping the role of both cohorts, and the coefficients of the best logistic regression models.

Supplementary materials
