# Supplementary material for: Machine Learning-Based Prediction of Impulse Control Disorders in Parkinson’s Disease From Clinical and Genetic Data
Source: IEEE Open J Eng Med Biol. 2022 May 27;3:96–107. doi: 10.1109/OJEMB.2022.3178295 (PMC9252337; doi:10.1109/OJEMB.2022.3178295)
Supplement: Supplementary materials [file supp1-3178295.docx]

**Reduction approaches**

Algorithms like logistic regression expect a fixed number of features as input. In order to deal with varying numbers of visits, we reduced all the previous visits into one “summary” visit using a convex combination. A convex combination is a linear combination such that the weights are all non-negative and sum to one. The weights indicate how much each visit contributes to this summary visit. A weight of 1 for the first visit means that the summary visit is simply the baseline visit, while a weight of 1 for the latest visit means that the “summary” visit is simply the most recent visit. One can also give uniform weights, so that each visit contributes equally to this “summary” visit, or higher weights to most recent visits if they are assumed to be more important than older visits.

Mathematically, if we have observations x at T time points:


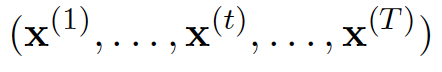


a convex combination is simply:


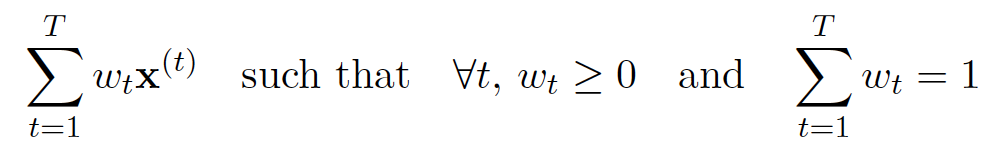


Each $w_{t}$ is the weight of time point t in this “summary” visit. The following table summarizes the different convex combinations that we investigated:


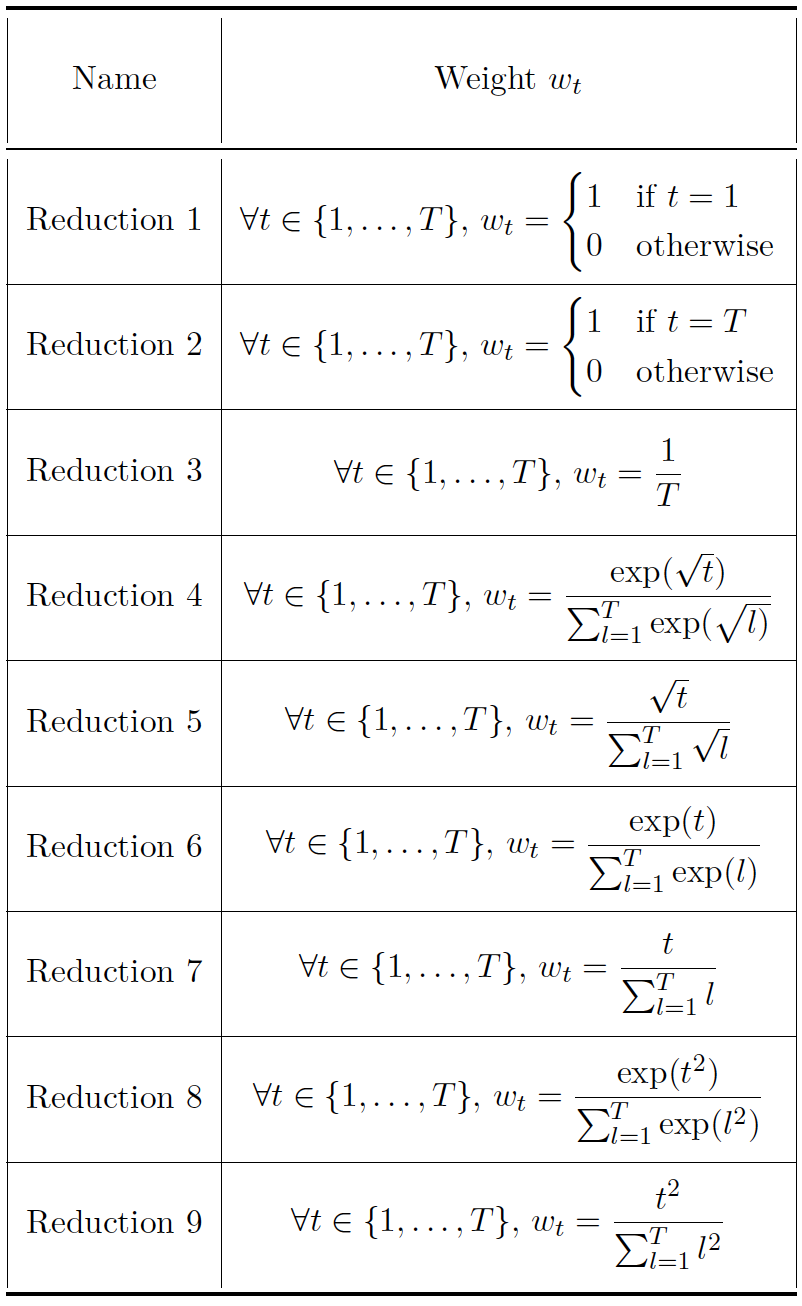


Reduction 1 corresponds to the baseline visit, while reduction 2 corresponds to the previous visit, and reduction 3 corresponds to the mean over the past visits.

**Captions for supplementary tables**

**Supplementary Table 1. Genetic variants included in the analyses.**

**Supplementary Table 2. Predictive performance of all the models.**

**Supplementary Table 3. Predictive performance of all the models using 10 repetitions of the cross-validation. Mean (± standard deviation) over the 10 repetitions are reported.**

**Supplementary Table 4. Predictive performance of all the models trained on DIGPD.**

**Supplementary Table 5. Predictive performance of all the models trained on DIGPD using 10 repetitions of the cross-validation. Mean (± standard deviation) over the 10 repetitions are reported.**

**Supplementary Table 6. Coefficients of the three main logistic regression models without genetic variants as input.**

**Supplementary Table 7. Coefficients of the three main logistic regression models with genetic variants as input.**
